# Control of Tibial Advancement by the Plantar Flexors during the Stance Phase of Gait Depends on Knee Flexion with Respect to the Ground Reaction Force

**DOI:** 10.3390/bioengineering11010041

**Published:** 2023-12-31

**Authors:** Reinald Brunner, Carlo Albino Frigo

**Affiliations:** 1 Department of Orthopaedics, Children’s University Hospital Basel, 4056 Basel, Switzerland; reinald-g-h.brunner@unibas.ch; 2 Department of Bioengineering, Medical Faculty, University of Basel, 4001 Basel, Switzerland; 3 Department of Electronics, Information and Bioengineering, Politecnico di Milano, 20133 Milan, Italy

**Keywords:** plantar flexion-knee extension couple, dynamic simulation, crouch gait, knee flexion in gait, plantar flexor control on tibial advancement

## Abstract

During the stance phase of a normal gait, the triceps surae muscle controls the advancement of the tibia, which contributes to knee extension. Plantar flexor weakness results in excessive dorsiflexion, and consequently, the knee loses this contribution. However, increasing knee flexion is also seen in patients with cerebral palsy who do not have plantar flexor weakness. We aimed to understand this mechanism through the use of a musculoskeletal dynamic model. The model consists of solid segments connected with rotatory joints and springs to represent individual muscles. It was positioned at different degrees of ankle plantarflexion, knee flexion, and hip flexion. The soleus muscle was activated concentrically to produce plantarflexion and push the foot against the ground. The resulting knee extension was analyzed. The principal determinant of knee flexion or extension associated with ankle plantarflexion was the position of the knee joint center. When this was anterior to the line of action of the ground reaction force (GRF), the soleus contraction resulted in increased knee flexion. The knee extension was obtained when the knee was flexed less than approximately 25°. The relation between joint angles, anthropometric parameters, and the position of the GRF was expressed in a mathematical formulation. The clinical relevance of this model is that it explains the failure of plantar flexor control on knee extension in patients with cerebral palsy, when increased knee flexion can occur even if there is a normal or plantarflexed foot position.

## 1. Introduction

Knee extension in standing and during the stance phase of normal gait depends on the control of tibial advancement by the triceps surae muscle, especially the soleus, which plantar flexes the foot. In standing, the plantar flexors tend to recline the tibia backwards by using the foot as a stable lever arm [1]. In this condition, the contribution of the knee extensor muscles to produce knee extension may not be necessary since the ground reaction force (GRF) produces an external extension moment at the knee, which can be counteracted by the posterior ligaments and joint capsule of the knee when it is hyperextended [1]. A similar condition occurs during normal walking. During the loading response, initial knee flexion is controlled by the eccentric contraction of the knee extensors, which then contract concentrically and extend the knee to about 15 to 20 degrees of flexion. Further knee extension during the stance phase is produced by the eccentric contraction of the triceps surae muscle [2,3]. The plantar flexor moment is transmitted to the forefoot, which acts as a lever arm and slows down tibial advancement while the pelvis and the center of mass move forward [4]. Overactive plantar flexors may produce hyperextension of the knee [5,6,7,8,9], as in cerebral palsy [4,7,10,11,12,13,14]. This mechanism is known as the plantar flexion-knee extension couple (PFKEC) [7,14]. It is the soleus muscle that has the potential to extend the knee [15]. Persisting plantar flexor overactivity may result in a structural deformity of the foot and ankle. Plantar flexor contractures can have the same effect as active soleus contractions and can compensate for triceps weakness, especially if the knee extensors are also weak, as in Duchenne muscle dystrophy [16]. Plantar flexor shortening leads to a condition known as equinus foot and reduces the functional lever arm at the ankle. Persistent foot loading in this position may eventually produce a ‘midfoot break’. This is a flat foot deformity that results in an ineffective lever arm and loss of plantar flexor control on the tibial advancement and thus on the knee extension.

**Figure 1:**
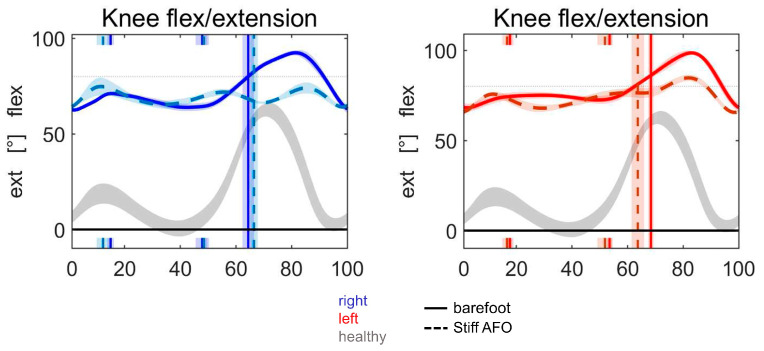
Failure of AFO treatment. Knee kinematic graphs from an instrumented gait analysis of a patient with bilateral cerebral palsy mainly affecting the legs. Although a knee extension was possible up to 25 degrees of flexion (knee flexion contracture), his dynamic extension during gait was limited to 70 degrees of flexion bilaterally. The bilateral treatment with stiff AFOs to gain better knee extension had no effect. Blue = right, red = left, grey = healthy, continuous = barefoot, dotted = stiff AFO.

The effectiveness of the plantar flexors depends on triceps surae strength and lever arm stability at the ankle and knee. Reduced triceps surae strength can result from overlengthened plantar flexors, poor muscle control, and/or muscle pathology. Lever arm efficiency, that is, the capability to transfer the plantar flexors’ contraction to the forefoot, decreases when the foot is excessively plantarflexed (equinus foot) or is affected by a severe foot deformity and instability. Both mechanisms can lead to crouch gait, a particular attitude characterized by an excessively flexed knee and excessive ankle dorsiflexion in the stance phase. This is frequently observed in children with cerebral palsy or when the plantar flexors are weak or paralyzed. When poor tibial control is due to plantar flexor weakness, a rigid ankle foot orthosis (AFO) can be applied to the affected limb. This prevents ankle dorsiflexion, restores foot stability, substitutes for plantar flexors’ weakness, and hence improves the control of tibial advancement and knee extension. There are situations, however, in which a flexed knee gait occurs even if there is minor or no dorsiflexion yielding, and the application of an AFO may not always help (Figure 1). Some individuals may have available knee and hip extension tested at passive clinical examination but continue to walk with flexed knees in stance, and this posture deteriorates over time. We wanted to investigate this apparent paradox, namely: sufficient range of motion is available at the hip and knee, yet an individual continues to walk with a bent-knee gait. The literature is relatively scarce on this phenomenon [10,11,12,13,14], and to our knowledge, no previous attempts have been made to identify the biomechanical principles behind it. So, we decided to exploit the potential of the musculoskeletal models to gain insight into the mechanism and the factors that make the PFKEC functional in certain conditions. Thus, this work consists of a dynamic simulation of different postural conditions to determine a synthetic formulation of the relationship between hip, knee, and ankle joint angles and anthropometric parameters. 

We hypothesized that the leg segments may move into a position with respect to the external GRF where the plantar flexors lose competence to control tibial advancement. This position could also explain the failure of AFO treatment, which aims to restore the lack of plantar flexor activity. As the position of the GRF is another factor apart from muscle activity, which may help with knee extension, the GRF was added to our considerations.

## 2. Materials and Methods

**Figure 2:**
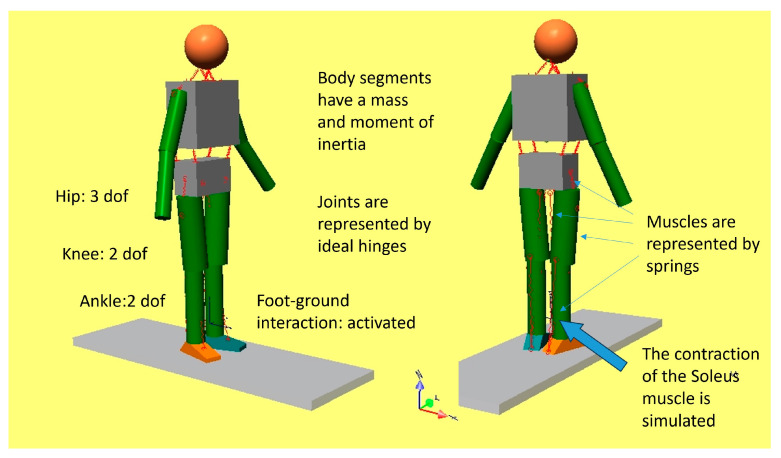
The multisegmental dynamic model.

A previously developed musculoskeletal dynamic model [17] was adapted for the present study. It consisted of a series of geometric solids representing the head, trunk, pelvis, thighs, shanks, and feet for the two lower limbs, and upper arms and forearms for the upper limbs, connected by different joints that allow their relative mobility (see Figure 2).

Spherical hinges between the pelvis and thighs permit the 3D rotation of the hip joints; two orthogonal revolute joints permit flexion/extension and internal/external rotation of the knee joints; and two revolute joints permit plantar/dorsiflexion and pronation/supination at the ankles. The main lower limb muscles were represented by linear springs, and muscle contractions were reproduced by changing the rest length of the springs, having predefined an artificial spring stiffness. The simulation platform was SimWise-4D (DST, Canton, MI, USA), which allowed the model to be animated either by inputting predefined kinematic variables (joint angles and trunk trajectory) or kinetic variables (forces and moments). The model configuration in space was defined by different hip, knee, and ankle joint angles, and the right foot was put in contact with the ground. To simulate the activation of the soleus muscle (SO), which was the object of our study, the stiffness of the corresponding spring was set to 100 N/m, and the other stiffnesses were set to zero so that the other springs did not produce any force. The resting length of the spring representing the SO was assumed to be the length measured in the initial posture. When the resting length of the spring was reduced by 20%, the spring produced a force that resulted in ankle plantarflexion. Since the model prevented penetration of the foot into the ground, a ground reaction force (GRF) was generated, which was applied at the forefoot. Thus, this reaction produced an acceleration of the various segments of the model and a change in the relative configuration as a consequence. The gravity field was removed to avoid the problem of balancing the gravitational forces since our objective was to understand the net effect of pushing against the ground. For the same purpose, the interaction of the contralateral foot with the ground was removed. In this condition, the resulting movements depended only on the ground reaction force produced by the SO muscle contraction and the dynamic coupling of the different segments. The standing upright position was simulated first. With all segments aligned and the foot flat on the ground, we analyzed the effects of the ankle plantarflexion produced by the SO contraction. Then we analyzed different initial postures by changing the hip and knee joint angles. The ankle joint was set in such a way that, in all conditions, the forefoot and not the heel were in contact with the ground. Since the knee joint was free to rotate, we could observe the effect of ankle plantarflexion on the knee joint in terms of knee flexion or extension. After considering the behaviour of the model under several initial conditions, we synthesized the mechanism of plantar flexor control on tibial advancement in a mathematical formulation.

## 3. Results

**Figure 3:**
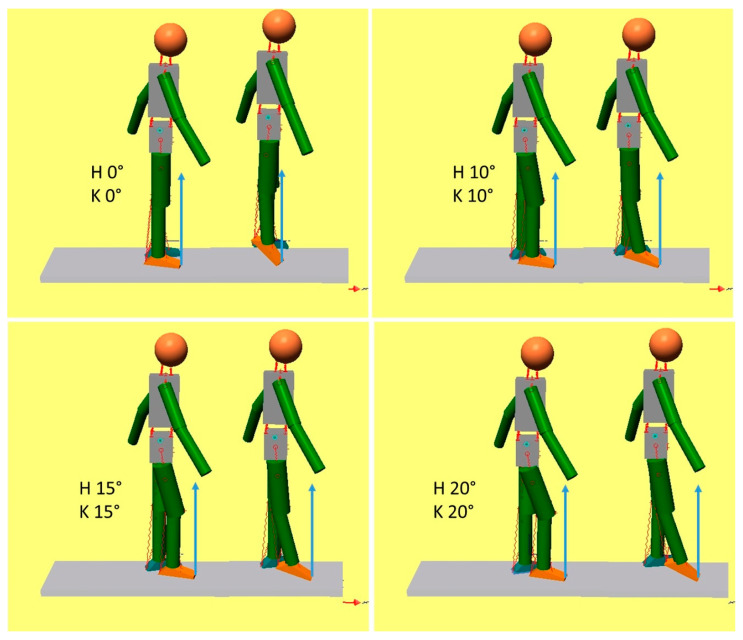
Plantarflexion extends the knee (plantar flexion-knee extension couple). In each panel, the initial position of the model is depicted on the left, and the position achieved after ankle plantarflexion is reported on the right. The values of the initial hip and knee joint angles are reported as H and K, respectively. A hypothetical ground reaction force has been drawn as applied at the tip of the foot to show its relationship with the knee joint.

Figure 3 is a graphic representation of the results obtained starting from different initial positions. The hip and knee joint angles put into the model are reported numerically in each panel. H 0 and K 0 indicate that the thigh and shank are aligned, as in the standing upright position. In all the other cases, the hip was slightly flexed at 10°, 15°, and 20°, and the knee was flexed by the same amount so that the foot was parallel to the ground. The initial ankle angle was 90°. After SO shortening, the plantarflexion of the foot occurred, and the model assumed the configuration reported immediately on the right in the same panel. In all the reported conditions, the plantar flexors produced a backward movement of the tibia, and this is evidenced by the hyperextension of the knee. Hyperextension was limited in our model to a maximum of 10° to prevent unnatural configurations; this value was promptly achieved in these simulations. As long as the knee is not flexed more than 20°, plantar flexor activity results in knee extension. In these situations, the GRF was placed in front of the knee rotation center.

**Figure 4:**
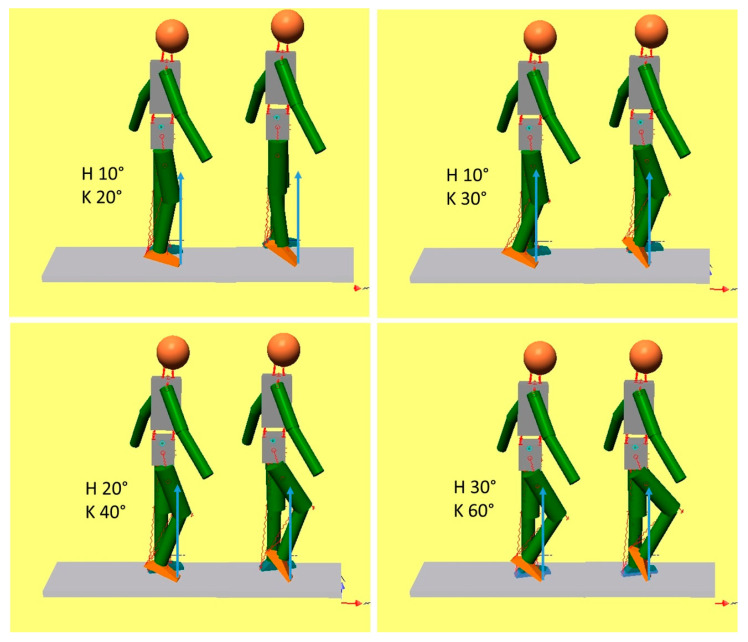
The GRF and the effect of the plantar flexors on knee extension. The legend is the same as in Figure 2. Here, the initial positions include increased knee flexion. It appears that, when the GRF lies posterior to the knee joint, increased knee flexion is produced instead of knee extension. In this case, the plantar flexors lose the competence to control tibial advancement.

In Figure 4, the simulations were conducted starting from initial conditions in which the knee joint was more flexed. Except for panel 1 (H 10°, K 20°), in all other conditions, ankle plantarflexion produces knee flexion instead of knee hyperextension. In all instances where knee flexion occurred, the GRF was located behind the knee joint center. Thus, it appears that the plantar flexor control on tibial advancement is not a constant phenomenon, but it may be determined by the position of the limb in relation to the GRF. If the knee is held flexed, the position of the GRF with respect to the knee joint center is relevant and predicts knee extension. However, if flexed 30° or more, it becomes difficult to place the GRF in front of the knee, and knee flexion becomes uncontrollable.

**Figure 5:**
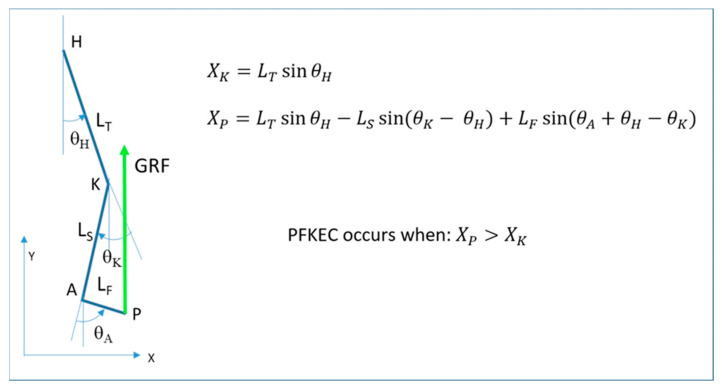
A simplified diagram of the lower limb and the GRF (left). The external knee moment is extensor in this case and would be flexor if the GRF was posterior to the knee (PFKEC: plantar flexor-knee extension couple).

Under a few simplifying assumptions, this mechanism can be formulated mathematically, as shown in Figure 5. The formula that predicts when the plantar flexors can control the tibial advancement is reported below. (*X_P_* is the horizontal coordinate of the point of application of the GRF; *X_K_* is the horizontal coordinate of the knee joint center.)

$$X_{K}=L_{T}\sin\theta_{H}$$

$$X_{P}=L_{T}\sin\theta_{H}-L_{S}\sin(\theta_{K}-\theta_{H})+L_{F}\sin(\theta_{A}+\theta_{H}-\theta_{K})$$

The plantar flexors can control the tibial advancement when $X_{P}>X_{K}$.

When the tibia is bent backwards, the contraction of the plantar flexors allows for a fast advancement of the GRF in front of the knee, which creates an early knee extension moment. If the tibia is inclined forward, the GRF remains behind the knee joint, and the knee is flexed. Such is what happens in crouch gait, but it may also occur in normal or plantarflexed foot positions if the knee is originally flexed. In this situation, the plantar flexors lose their knee-extending effect.

The simplifying assumptions adopted in our mathematical formulation are that the GRF is applied at the forefoot and is vertical, which means negligible horizontal components and no influence from the contralateral foot contact.

**Figure 6:**
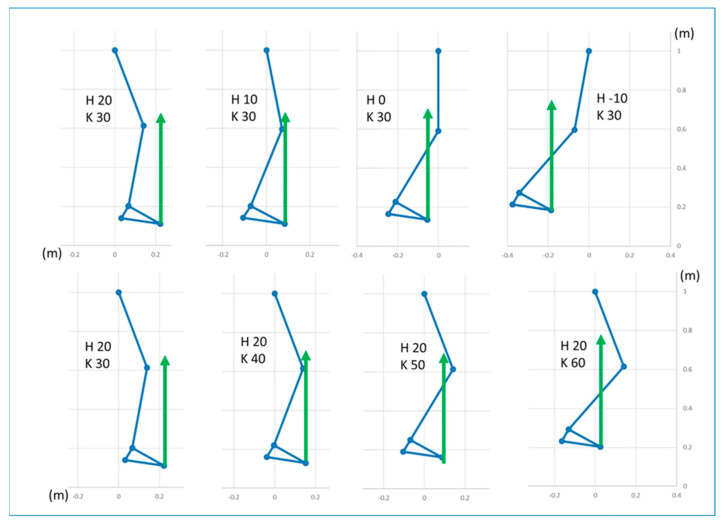
Examples of conditions enabling the plantar flexors to control tibial advancement (the GRF in front of the knee joint center) or preventing it (the GRF behind the knee joint center). **Upper** row: different hip joint angles, knee joint flexed at 30°; **lower** row: different knee joint angles, hip flexed at 20°. In these examples, the foot longitudinal axis is always oriented 30° towards the floor (foot–floor angle).

Figure 6 shows some of the conditions that enable plantar flexor control on tibial advancement and conditions when it does not occur.

**Figure 7:**
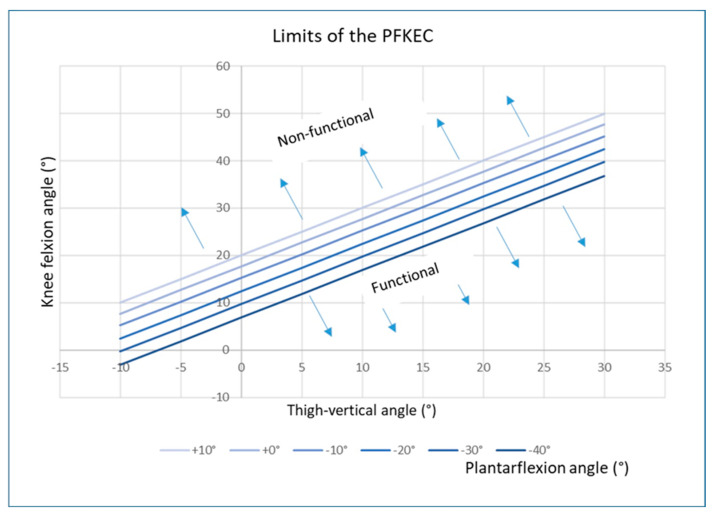
Fields of angle values that define the competence of the plantar flexors to control tibial advancement (functional or non-functional). They are separated by a line that depends on the ankle plantarflexion (see explanation in the text).

Figure 7 demonstrates the limit angles between the occurrence (functional) and non-occurrence (non-functional) of plantar flexor control on tibial advancement.

The separation of the two fields is represented by the straight lines reported in the figure. They were obtained by implementing the equations reported in Figure 3 and solving for *X_P_ = X_K_*. For each value of *θ_H_*, the limit value of *θ_K_* was computed for different values of ankle plantarflexion *θ_A_*. This helps to understand how the ankle plantarflexion affects the limit of the knee flexion angle when the hip angle (thigh-vertical) has a predefined value. For example, looking at Figure 6, for a thigh-vertical angle of 20° (a value corresponding to a typical crouch gait attitude), the limits of the knee flexion angle at which the plantar flexors lose their competence to control tibial advancement change from about 40° when the ankle was 10° dorsiflexed to approximately 27° when the ankle was 40° plantarflexed. This means that the plantar flexion-knee extension couple is still effective for relatively high knee flexion if the ankle joint is dorsiflexed or slightly plantarflexed, but needs a less flexed knee joint to be functional if the ankle is largely plantarflexed.

## 4. Discussion

Adequate knee extension in the stance phase of normal gait is obtained not only by the activity of the quadriceps but also by the plantar flexor muscles, which control the forward rotation of the tibia. By holding the tibia back, the forward movement of the center of mass at trunk level extends the knee. However, the same mechanism could also flex the hip. Thus, control of hip extension is required. In pathological cases, this mechanism may not occur for various reasons. Plantar flexor control on the tibia requires sufficient muscle strength, and the foot is the lever arm of action for the plantar flexors. Hence, the plantar flexors’ weakness can be one reason that may cause the tibia to rotate forward early after load acceptance. Causes of muscle weakness are pathologies of the muscle tissue, loss of innervation known as paresis, and plantar flexor overlength. Another reason is the affection of the foot as a lever arm. Foot malrotation or loss of intrinsic foot stability causes difficulty in transferring the load to the forefoot (lack of lever arm). The resulting situation is characterized by increased dorsiflexion associated with knee and hip flexion in the stance phase of gait and is known as crouch gait [18,19]. However, an important plantarflexion shortens the lever arm. While in a normal standing or heel-toe gait, the whole foot is loaded and the GRF moves from the rear to the forefoot, standing on the toes involves only the forefoot as a loaded area. In order to maintain balance, patients usually flex their hips and knees. In this situation of flexion, more activity from the extensor muscles at the knee and hip joints is required. The hamstrings are part of the hip extensors [18]. Overactive muscles generally become structurally short if they are not regularly stretched. In the case of crouch gait, the plantar flexors and the hamstrings are mainly concerned. Furthermore, the constant lack of knee and hip extension leads to flexion contractures of these joints over time. Knee flexion is thus explained by plantar flexor incompetence to control the tibia and, later on, by secondary deformities of muscles and joints. The situation is true not only for standing but also for the stance phase of gait. In cases of plantar flexor incompetence, stiff AFOs are usually applied to regain control of the tibia and improve knee extension. The AFO aims at correcting the lever arm dysfunction of the foot by stabilizing and redirecting the foot and by substituting the plantar flexor activity by holding the ankle stiff. Once structural deformities have developed, such treatment cannot be efficient anymore. Clinically, however, there are CP patients who have a normal or slightly plantarflexed foot, sufficient muscle strength, and no secondary deformities but still walk with flexed knees and hips, and they even do not respond to AFO treatment.

An example is shown in Figure 1. We therefore searched for a mechanical explanation. In addition to the plantar flexors, the position of the GRF is essential to understanding knee extension. A position in front of the knee joint rotation center creates an extension moment, which helps with knee extension. As plantarflexion leads to forefoot load, the point of attack for the GRF is located in the forefoot. The GRF was thus positioned relatively anteriorly compared to normal. This study was undertaken to understand the role of the plantar flexors in this situation, which led us to consider a third reason for the failure of knee extension control by the plantar flexor muscles. This is related to the degree of knee flexion when the load is on the forefoot. Our considerations derive from the use of a musculoskeletal model and explain this mechanism based solely on biomechanics. We have shown that the effectiveness of the plantar flexors to control tibial advancement depends on the position of the leg segments in space and their relationship to the GRF. If the knee joint is sufficiently flexed but the GRF remains in the back of the knee rotation center, ankle plantarflexion may produce additional knee flexion instead of extension. In contrast, if the GRF moves in front of the knee joint center, the knee-extending effect of the plantar flexors by controlling tibial advancement can still be functional. The clinical consequence is that, with increased knee flexion and a lack of competence of the GRF to extend the knee, the knee extensors have to sustain the moment produced by the GRF for the whole time in which the knee is flexed. In addition, if the gastrocnemii contribute to the plantarflexion together with the soleus, the knee extensors have to also counteract the flexion moment produced at the knee by these double joint muscles. We were able to define mechanical conditions that explained the knee flexion and the lack of response to treatment. A formula is provided that offers the possibility of calculating when the plantar flexor control on tibial advancement might become non-functional. The model shows that an angle of approximately 25° of knee flexion is the limit in a normal upright posture, with the GRF crossing the knee rotation center. It is possible, however, to accommodate knee flexion up to 40° and still achieve plantar flexor control on tibial advancement if the GRF is positioned in front of the knee joint center. In this case, the trunk is bent forward and the hip is flexed to position the center of mass forward. However, knee flexions greater than 40° lead to failure of the plantar flexor contribution to knee extension in spite of the anterior position of the GRF.

This dependence of the plantar flexor competence to control tibial advancement from the amount of knee flexion and the GRF position helps to explain the role of knee extensors in normal gait as well: after the initial weight acceptance, the knee extensors extend the knee as much as is required to align the lower limb segments in a position that enables the plantar flexors to control the tibial advancement. Then the extensor moment can be reduced because the plantar flexors contribute to the knee extension. If the initial knee extension is inadequate, the plantar flexor control on tibial advancement is non-functional, and the knee remains persistently flexed. This position results from the knee extensors being unable to produce sufficient extension during the loading phase. This also explains the failure of an AFO to extend the knee in patients who have excessive knee flexion. To recover from the lack of a plantar flexor component for knee extension, patients can lean their trunk forward to move the GRF forward and reduce the flexion moment at the knee. This posture, however, depends on the effectiveness of the hip extensors, including the hamstrings. Since these bi-articular muscles produce a flexion moment at the knee, it appears that the knee extensors still have to sustain an increased knee moment. Failure of plantar flexor control on tibial advancement and the possible compensation strategy can both cause overloading of the knee extensors and result in an elongation of the patella tendon and a high-riding patella. The contraction of the hamstrings in the presence of a flexed knee may also exacerbate the gait deformity and contribute further to overloading the knee extensors. These mechanisms explain a deterioration in crouch gait even though the plantar flexors may remain functional or are substituted by orthotics.

Patients may use different strategies to optimize an efficient gait. The individual amount of knee flexion, plantar flexor strength, and control of the center of mass introduce a wide range of variables. However, our model provides biomechanical principles to explain what may be observed in clinical practice. It provides new guidance to the point beyond which excessive knee flexion may need to be treated to ensure efficient plantar flexor control on the tibia.

One drawback of this study is that our model did not use data from patients. Musculoskeletal modeling with information on possible muscle activity distribution might provide better insight, but the altered muscle structure in patients with cerebral palsy or spina bifida could be an issue. Our reason for using the model described in this study was because we wished to isolate the effect of plantarflexion on the hip and knee and exclude confounding effects from other muscles on knee extension. The knee flexion angles reported as limits between functional and non-functional plantar flexors to control tibial advancement may differ between subjects and depend on many factors. Our mathematical formula takes into account the most relevant geometrical factors, i.e., the length of the lower limb segments, the inclination of the thigh in relation to the vertical line, and the ankle plantarflexion. Although our results are not derived from a patient, the simulations closely represent the situation observed clinically in mid-stance during gait.

The clinical significance of this paper is that it provides a possible explanation of the failure of plantar flexor control on tibial advancement and a theoretical range of angles at the hip and knee when this might occur. This needs to be confirmed in patients but has the potential to guide clinical decisions for optimizing gait and orthotic management.

## 5. Conclusions

This paper provides a possible explanation for when the plantar flexors may fail to control tibial advancement during the stance phase of gait and, as a consequence, the knee extension associated with plantarflexion (the plantar flexion-knee extension couple, PFKEC) does not occur. From our biomechanical analysis, the combination of hip, knee, and ankle joint angles together with anthropometric parameters concur to make the PFKEC ineffective. By just accepting a few simple assumptions, the key point appears to be the relative position of the line of action of the GRF in relation to the knee joint center. When it is in front of the knee joint center, the moment produced by the GRF is extensor, and so there is a tendency to extend the knee; when it lies behind the knee joint center, the tendency will be to flex the knee. Once this principle is ascertained, the relationship between joint angles and segments’ length can be formulated mathematically, as represented in Figure 5. Based on these considerations, the reasons for the deterioration of flexion during gait, crouch gait, or knee flexion gait deformity can be understood. The model also helps to understand why treatment with an ankle foot orthosis aimed at extending the knee during gait may be ineffective. Our study provides a theoretical range of angles at the hip and knee where the plantar flexors may fail and also provides an estimate of the degree of knee joint deformity that could be tolerated.

## Data Availability

Data are available upon request to the authors.
